# Monthly Summary

**Published:** 1896-07

**Authors:** 


### Monthly Summary.

COHESION AND ADHESION.—The dispute about the definition of these words crop up in dental societies and classes in colleges very frequently, and it is astonishing to see the pertinacity with which some people will cling to obsolete or preconceived opinions. The following definitions may be relied upon and are given upon the best authority: Cohesion is that force by which molecules of a body are held together, and adhesion is that force by which similar or dissimilar bodies or surfaces adhere or stick together. Adhesion is cohesion in mass. Obsolete definitions of these terms are as follows: That cohesion must be between like molecules; that adhesion is between dissimilar bodies or surfaces.

TOOTH IN HER LUNG.—Mrs. Patrick Pierson, who lives with her husband at 66 Division street, Elizabeth, N. J., went on Saturday, Oct. 26 last, to a dentist in this city to have a tooth extracted.

Her husband declines to say who the dentist is, as he does not want to injure his business. Mrs. Pierson wanted to have a wisdom tooth extracted and she took laughing gas.

When she recovered her senses she felt a choking sensation, but at that time she ascribed it to the effects of the gas. A few minutes later her breathing became affected much as though she had been suffering with asthma.

Her condition became worse on Sunday, and on Monday she was so ill that a physician, Dr. Mack, was called.

He pronounced the disease to be pneumonia, and treated Mrs. Pierson for it. Then the pneumonia symptoms disappeared, and symptoms of pleurisy developed.

These were followed by other peculiar symptoms, and the doctor became puzzled. He seemed to be able to stop every disease that seemed to develop, but he failed to cure the patient after six weeks of varying treatment. Dr. Mack became convinced that some unusual affection had attacked Mrs. Pierson's lung, as she coughed almost incessantly, and he had her removed to the hospital.

A consultation of physicians of the staff was held, and it was decided that the only method of treatment for a case of this mysterious kind was to open the lung cavity. It was decided at the same consultation that Mrs. Pierson was too weak to stand a surgical operation.

Mr. Pierson was told, he says, that his wife was incurable.

He then had her taken back to their home and called Dr. J. P. O'Reilly to attend her. This physician kept the woman alive for three weeks, but she did not get any better, and at the suggestion of his sister-in-law Mr. Pierson summoned Dr. E. R. O'Rellly. The latter had hopes of saving Mrs. Pierson, and began a course of treatment that apparently increased the violence of the woman's coughing. The patient had grown extremely weak from continual coughing, and her friends expected her to die at any time.

On Friday last she had an extremely severe fit of coughing. In the midst of it she said she felt something coming up in her throat. Finally she coughed up something.

The something was hard, encysted, and two prong-like projections from it attracted the attention of her husband, who discovered that they were the roots of a tooth. It was, in fact, the wisdom tooth of Mrs. Pierson, which the dentist had extracted while she was unconscious and which he must have allowed her to draw into her windpipe. Since she coughed it up she has rapidly improved.

The physicians were astonished when they learned the cause of her illness The explanation given as to why the tooth was not coughed up before is that one of the roots was curved at the end in the shape of a J and had become hooked in the membrane of the lung.

---

REPAIRING BROKEN DOWN TEETH.—By Dr. D. Murlless, Holyoke, Mass.—Now, if we consider an incisor with a third, a half, or even more decayed and broken down from the proximal and cutting edge, taking away a large corner of the tooth, and in many cases containing a live pulp, and when we reflect on the troublesome consequence of death of the pulp, or even pulp irritation, which is very likely to follow filling, we see that it is imperatively demanded that the effort at pulp-conservation be made. Such a tooth as I have described can be filled and restored to its original size and shape by first filling with some plastic, and then putting on a gold cap or crown, with an opening in its face of such shape and size as will just cover the margin of the cavity, having but very little more gold in view than would be seen if the tooth had simply been filled.

There are many advantages in this method, as teeth can be saved that it would be nearly impossible to preserve by other treatments; for example, many times we find the molars of the lower set lost, and persons in such a condition in using their teeth bring the lower incisors against the lingual side of the uppers. Where they have been thus used for some time the lower teeth will be worn on the cutting edge and shortened, and the lingual surface of the uppers will be worn by abrasion, so much so that the under side of the uppers will often be worn away, forming a shoulder at the neck of the tooth, and occasionally the labial surface will be worn thin. In such cases there is no way to retain a filling, but the tooth can be backed up with cement, and a cap, such as I have described, be telescoped over it. By this means the cutting edge is thoroughly protected, the whole tooth bound and held firmly together, and we may say it is as strong and serviceable as ever, and needs no more care than if it were perfectly sound. I had successfully used this method in my own mouth some time before I saw it spoken of anywhere.—*Dental Digest.*

---

THE ADVERTISING DENTIST.—The value of the Code of Ethics lies largely in the restriction it places on the advertising of self. If the merchant or manufacturer desires the public to know of an article that he may have to sell, he advertises the same, and is perfectly justified in doing so. Then why, you ask, is not the professional man justified in doing the same? I answer, because in the one case the man advertises his goods, while in the other he extols himself. What can be more disgusting to the true professional spirit than the man who is guilty of this offense?—Abstract from the annual address of the President of the Mississippi Dental Association, 1896, by Mrs. J. M. Walker.—*Items of Interest.*

---

WHAT WE NEED.—What the man of to-day needs most is not athletics in a gymnasium, but plenty of fresh air in his lungs. Instead of a quantity of violent exercise that leaves him weak for several hours afterward, he needs to learn to breathe right, stand right and sit right. The young man or young woman who starts on a career of training, and keeps it up year and year, just at the time when the body has a great deal of its own natural work to do and wants to do it, may make up his or her mind that beyond a showy and superficial development of muscle and strength, all this training in after life is going to count against them.—*Annals of Hygiene.*

DENTAL HEMORRHAGE.—By chance I tried carbolized rosin in hemorrhage. I do not pronounce it the "greatest styptic in the world," but I do claim it has never failed me.

As the result of experiment, this is the formula I prefer:

| | | |
|---|---|---|
| R. | Pulverized rosin (common) - - | ʒ iv |
| | Carbolic acid (65 per cent) - - - | ʒ jii |
| | Chloroform - - - - - | ʒ ji |
| M. | | |

Make a short, thick cotton rope, larger than the wound to be treated, moisten the end well with the compound and plug the cavity tightly. The bleeding will cease almost as if by magic.

Its adherence to the tissue in which it is placed makes it unlikely to be forced out of its place by the pressure of the blood.

After a wound caused by extraction of a tooth has been plugged with cotton for four or five hours, considerable pain ensues, caused by the pressure of the cotton; so I advise patients to remove cotton after a lapse of a few hours.

Besides being so valuable a styptic, carbolized rosin is almost a specific for toothache if caused by exposed pulp.

[Would not carbolized rosin be good also as a covering for exposed, or nearly exposed, pulp before cement filling; also for sensitive dentin?—ED. ITEMS.]

A platinum loop placed in a tooth socket and used as an electric cautery will often stop a hemorrhage.—Dr. J. Van Pelt Wicks, in *Items of Interest*.

---

CARBOLIC ACID.—In *The Polyclinic* Dr. Oscar H. Allis, of Philadelphia, has contributed an article worthy of wide-spread circulation. He says the use of carbolic acid in full strength upon the fresh tissues, raw surfaces, etc., causes the formation of a protective albuminate, a condition which renders further absorption impossible.

The same takes place when the strong acid is applied to a raw burned surface. It is not claimed that an aqueous dilution is safe when applied extensively to raw surfaces; on the contrary, the more dilute the more dangerous. In a case of washing out the thorax in the treatment of purulent pleurisy, the late Roger Keys, a most careful and judicious physician, came near losing a patient from absorption of the dilute acid.

"It will strike many of you with astonishment when I say that it would be safer to pour a gallon of pure carbolic acid into a purulent thoracic cavity than to pour in a gallon of water into which a single ounce of carbolic acid has been placed. I will go even further, and say that excess of the strong acid in a cavity such as an abscess cavity, or upon exposed tissues, as a burn or a fresh wound, does no harm, while excess of a dilute solution, if left in a cavity or used over an extensive raw surface, will be promptly followed by dangerous, if not fatal, toxic effects."

---

NO TOOTHACHE IN THAT FACTORY.—There is a large manufacturing establishment on the West Side which employs a dentist to examine the teeth of all applicants for work. If a tooth has a cavity, it must be filled, or, if it is too far gone, it must be pulled. The dental work is, in most cases, done at the expense of the factory, and has proven to be wise economy. Little time is lost on account of toothaches. Teeth of employes are examined at regular intervals, whether they are giving their owners any trouble or not.—*Chicago Times-Herald.*

---

INSOMNIA—The utility of heat as a remedy for sleeplessness can scarcely be overestimated—particularly in the form of hot water. Insomnia is frequently overcome by the persistent use of hot foot baths, and simple hot water as a drink at bedtime. Sleeplessness is commonly caused by overfullness of the bloodvessels of the head; the bathing of the feet draws the blood from the head, the hot drink distributes the gases of the stomach, and gives one a sense of general comfort.—*Items of Interest.*

### CHRONIC GASTRITIS OF LONG STANDING, WITH PERIODIC ATTACKS OF MIGRAINE.

### REPORT OF A CASE.

BY GEO. A. CURRIDEN, M. D., CHAMBERSBURG, PA.

(Published by the *Medical Summary* of Philadelphia, Pa., for March, 1896.)

The herewith reported case is one of double interest, inasmuch as the patient has been under my care for a number of years, and previous to the commencement of the present treatment, I have been unsuccessful in affording much relief or preventing the recurrence of the frequent and periodic attacks of migraine, to which she had been more or less subject to since early womanhood. The cause of which I could not accout for more than "a habit long continued," aggravated by gastric catarrh.

The history of the case is briefly as follows: Mrs. A., age 55, since early womanhood has been subject to periodic attacks of migraine at intervals of two, three or four weeks, but seldom free from them for long intervals.

An attack comes on by general malaise of usually a day's duration, repugnance of food or drink, marked drowsiness, much depression with request for rest and quiet, followed by complete physical prostration, dull frontal headache, which the least noise or disturbance makes the more intense, invariably accompanied by violent and frequent attacks of vomiting and retching, inability to retain any food or nourishment of any kind, retention of bowels, often cold sweats, pulse somewhat slow and weak and small in volume. This condition lasting usually two days, followed by gradual cessation of symptoms.

During the whole period of usually four or five day's duration, she is unable to take nourishment of any kind, remains constantly in bed, and desires only complete rest and quiet. The previous treatment has been so varied and on so many different plans, that I refrain from mentioning them.

Two years ago I was able to prevent an attack for over two months by the use of strychnine in 1-20 grain doses t. i. d. with careful diet and artificial digestive.

In May, 1895, I put her on Charles Marchand's "glycozone" in teaspoonful doses well diluted t. i. d., using this as all other previous remedies experimentally; she commenced to improve much in general health, an unusually good appetite, without the previous distressing symptoms following, a more regular movement of the bowels, freedom from headache, and in every way a decided improvement; this improvement and enjoyment of good health lasted during continuation of above treatment for over three months. Unknown to me she stopped taking the glycozone, thinking herself perfectly well. In a few weeks had a return attack, milder and devoid of gastric distress. A similar attack two months later, both of which occurred some weeks after stopping the above described treatment, and I might say caused by imprudence in diet.

The conclusion come to, in this case is that the headache is sympathetic, that the stomach becomes acutely inflamed by its inability to naturally and properly perform its functions, and responds to the call of nature to unload itself, and thus secure for a time rest, that the use of glycozone has corrected the existing gastritis and by so doing has removed the primary cause of these many years of suffering.

---

DR. JOHN C. STORY, Dallas, Texas, editor of the *Texas Dental Journal*, assumes that "calcareous deposits about the teeth are much lessened, if not entirely suspended" during the period of pregnancy. Such an assumption is contrary to all observation has taught us. We daily see salivary calculus and conditions of pyorrhea in mouths of pregnant women who formerly were free from such irritation. The mouth at this period requires more than usual attention and receives less, and the result is seen daily in increase of caries, in fermentative processes and calcareous accretions.

---

AS A RULE, bad breath arises from a disordered stomach, and in such cases, when once the bowels have been regulated by a course of mild aperients, the trouble will vanish. It may, however, be caused by decaying teeth, or by neglect of the teeth altogether.

